# Electrophysiological Correlates of Subitizing in Healthy Aging

**DOI:** 10.1371/journal.pone.0131063

**Published:** 2015-06-22

**Authors:** Silvia Pagano, Elisa Fait, Alessia Monti, Debora Brignani, Veronica Mazza

**Affiliations:** 1 Center for Mind/Brain Sciences (CIMeC), University of Trento, Trento, Italy; 2 IRCCS Istituto Centro San Giovanni di Dio Fatebenefratelli, Brescia, Italy; 3 Department of Psychology and Cognitive Science, University of Trento, Trento, Italy; University of Groningen, NETHERLANDS

## Abstract

To understand the nature of age-related changes in enumeration abilities we measured two ERP responses -N2pc and CDA, associated respectively to attentive individuation and VWM- and posterior alpha band (8-15 Hz) event-related desynchronization (ERD), traditionally linked to enhanced target processing. Two groups of old and young participants enumerated a variable number (1-6) of targets presented among distractors. Older participants were less accurate in enumerating targets. ERP results in old participants showed a suppression of N2pc amplitudes for all numerosities, and a decrease in CDA only for the largest set (4-6 targets). In contrast with the pattern for young adults, time/frequency results on older adults revealed neither a modulation of alpha oscillations as a function of target numerosity, nor an effect of ERD lateralization. These patterns indicate that both attention and working memory contribute to the age-related decline in enumeration, and point to an overall decrease in the activity of the visual areas responsible for the processing of the hemifield where the relevant objects are presented.

## Introduction

The ability to process multiple objects simultaneously is essential to perform many tasks in everyday life, such as avoiding cars while crossing a street or counting the apples on a market stand. Therefore, evaluating how this ability changes throughout lifespan is important to assess the integrity of the cognitive functions in aging.

Previous behavioral studies have shown that there is a decline in the ability to process multiple objects simultaneously during aging [1–3]. Such decline has been assessed using visual enumeration tasks, in which participants are required to count a varying number of targets. A typical feature of enumeration tasks is represented by the “subitizing phenomenon” [4,5], namely the fact that participants are fast and accurate when they enumerate a relatively small (up to four) number of objects, whereas their reaction times and error rates increase dramatically for larger quantities. The subitizing effect is preserved in aging when the to-be-enumerated items are presented in isolation, but it is reduced when these are presented among distractors [3], leading to an overall decline in performance with respect to young adults [3,6]. These results have been interpreted as evidence of reduced attentional resources in aging, and are in line with the results of recent studies (e.g. [7,8]) showing that attention is generally impaired during aging.

However, multiple object processing does not exclusively depend on attentional resources. Recent studies on young adults [9–12] have pointed out that there is also a visual working memory (VWM) component involved in multiple object processing. In particular, these studies have shown that at least two mechanisms are implicated: an early attentional mechanism, associated with the simultaneous individuation of a small set of target quantities, and a late VWM component related to the active maintenance of the individuated objects. Therefore, age-related changes in the enumeration function could also be attributed to a decline of the functioning of VWM procedures. This hypothesis is also supported by a large corpus of ERP data [13–15] and oscillatory measures [16], indicating larger recruitment of resources in aged people during working-memory tasks.

The aim of the present study is to understand which stage of multiple object processing is mainly involved in the age-related decline during enumeration. We addressed this issue by recording EEG in old and young participants while they enumerated a variable number of uniquely colored targets (1–6) presented among distractors. Specifically, we focused on two ERP components previously associated to target individuation (the N2pc component) and VWM procedures (the CDA component), and on the posterior alpha oscillatory patterns related to visual perceptual processing.

The N2pc (N200 posterior contralateral) is a response elicited at around 200 ms post stimulus onset in posterior electrodes contralateral to a lateralized target presented among distractors [17,18], and it has been associated with attentive target selection. N2pc has been found in a variety of visual tasks involving both single [17–21] and multiple target processing [9,10,12,22]. In the latter case, the amplitude of N2pc increases as a function of target numerosity, reaching a plateau around three/four items. This result has been interpreted as evidence that N2pc specifically tracks the simultaneous individuation of a limited set of relevant objects.

The CDA component (Contralateral Delayed Activity, also named Sustained Posterior Negativity, [23,24]) is a lateralized sustained activity emerging at around 400 ms post-stimulus onset in posterior sites when multiple objects must be encoded and maintained in VWM [25,26]. While early studies have measured this component in delayed match to sample tasks during the retention period [24–26], or during multiple object tracking [10,27], recent studies have demonstrated that CDA is visible also during visual enumeration [11,12]. This suggests that the VWM procedures reflected by this component are involved in a variety of tasks requiring simultaneous processing of multiple objects. Similar to N2pc, CDA is also modulated by target numerosity, increasing as the number of targets increases up to a maximum of three/four targets.

Both N2pc and CDA have been recorded in aged participants during processing of a single target [28,29] and in multiple object processing tasks like delayed match-to-sample judgments [30,31] and multiple-object tracking [15]. These studies have found a suppression of the CDA in the delayed match to sample task and a suppression of the N2pc component both in single target detection and during multiple object tracking for the old group, meaning an age-related decline in both individuation and VWM. However, whether both processes would decline in visual enumeration tasks is currently unknown. If aging impairs the ability to individuate multiple targets during enumeration, we should expect to find a suppression of the N2pc component in the old group, similarly to the case of multiple object tracking tasks [15]. In contrast, if the age-related decline in enumeration is due to impairments in active maintenance of the target elements in visual working memory, we should observe a suppression of the CDA component in the old group, as previously found in working memory tasks [30].

In addition to the ERP measures, we also focused on the pattern of alpha (8–15 Hz) oscillatory activity (via Event-related synchronization/desynchronization, ERS/ERD [32,33]) to further understand the nature of enumeration changes in aging. Alpha band is a prominent cortical rhythm measured from posterior areas, and typically associated to preparatory effects relative to upcoming visual stimuli [33–37]. Previous studies on change detection [38] and on sequential enumeration [39] in young individuals have indicated that the amplitude of alpha post-stimulus oscillations is modulated by the numerosity of the elements to be processed, suggesting a link between multiple object processing and alpha frequency in visual areas. Thus, we expected to find a numerosity-related modulation of alpha activity in young participants in our study where simultaneous rather than sequential stimuli had to be enumerated. The use of lateralized targets in the present study allowed us to disentangle whether any effect of numerosity in enumeration tasks would be specifically related to the processing of the elements in the relevant (target) hemifield or whether distracter suppression in the irrelevant hemifield drives the numerosity-related modulation of alpha band (see [40]). This aspect was additionally useful for the comparison with the old group, in order to evaluate the link between changes in enumeration abilities and the processing of the relevant versus irrelevant hemifield.

## Methods

### Participants and Ethics

The effective samples include 18 young adults (mean age 23.3, female = 16) and 18 old participants (mean age 69.7, female = 9), who volunteered for the study after giving their written informed consent. All the procedures were approved by the local ethics committee of University of Trento and were conformed to the WMA- Declaration of Helsinki for research involving human subjects. All participants had normal or corrected-to-normal sight and reported no colorblindness. Participants with a history of neurological, psychiatric, or epileptic disorders were excluded from the sample. Other exclusion criteria were chronic use of sleep-inducing or psychopharmacological medications, left-handedness and head dermatitis.

### Neuropsychological testing

The group of old participants underwent a session of neuropsychological testing scheduled about a week before the EEG recordings. In the neuropsychological session participants performed a battery of tests, for which an Italian standardized version was available, aimed at assessing their cognitive efficiency, mainly related to their visuo-spatial and attention abilities. The results of the neuropsychological tests are summarized in Table 1.

**Table 1:** Demographic and neuropsychological data relative to old participants.

	Subjects’ score range	Mean (SD)	Median
**Demographic data**			
Age		69.8 (3.8)	
Education		12 (2.9)	13
**Neuropsychological tests**		Raw scores	
MMSE^a^	27–30	28.6 (1.08)	29
RCPM 47	25–36	32.7 (2.9)	33.5
Attentive Matrices (Visual Search)	46–59	54.7 (3.6)	55.5
TMT A	21–54	37.1 (9.5)	35
TMT B	67–214	112.6 (42.2)	100.5
TMT B-A	22–187	75.5 (41.5)	66.5
Stroop reaction times	15–39.5	24.3 (7.3)	24.2
Stroop errors	-1.5- +5	0.7 (1.7)	0
Phonemic fluency	23–59	38.8 (10.9)	41.5
Digit Span Forward	4–8	5.7 (0.9)	6
Digit Span Backward	3–8	4.6 (1.1)	4
ROCF-copy	31–36	34.5 (1.4)	35
ROCF-recall	7–25.5	17.4 (5.8)	18
RAVLT-immediate recall	27–65	47.6 (10.6)	46.5
RAVLT-delayed recall	6–15	11.5 (2.5)	11.5
Geriatric Depression Scale	0–14	3.6 (3.6)	2

The table represents the range of uncorrected values obtained from each test in our sample (subjects’ score range), the mean test scores (SD in parentheses), and the median values.

^a^Abbreviations: Mini Mental State Examination-MMSE [49], Raven’s Coloured Progressive Matrices-RCPM 47 [50]; Attentive matrices [51], Trail Making Test A and B [52], Stroop Test [53], Verbal fluency with phonemic cue [54], Digit span forward and backward [55], copy and recall of Rey-Osterrieth complex figure-ROCF [56], Rey’s Auditory Verbal Learning Test-RAVLT (15 items), [57] and Geriatric Depression Scale [58]

### Stimuli and procedure

**Fig 1:**
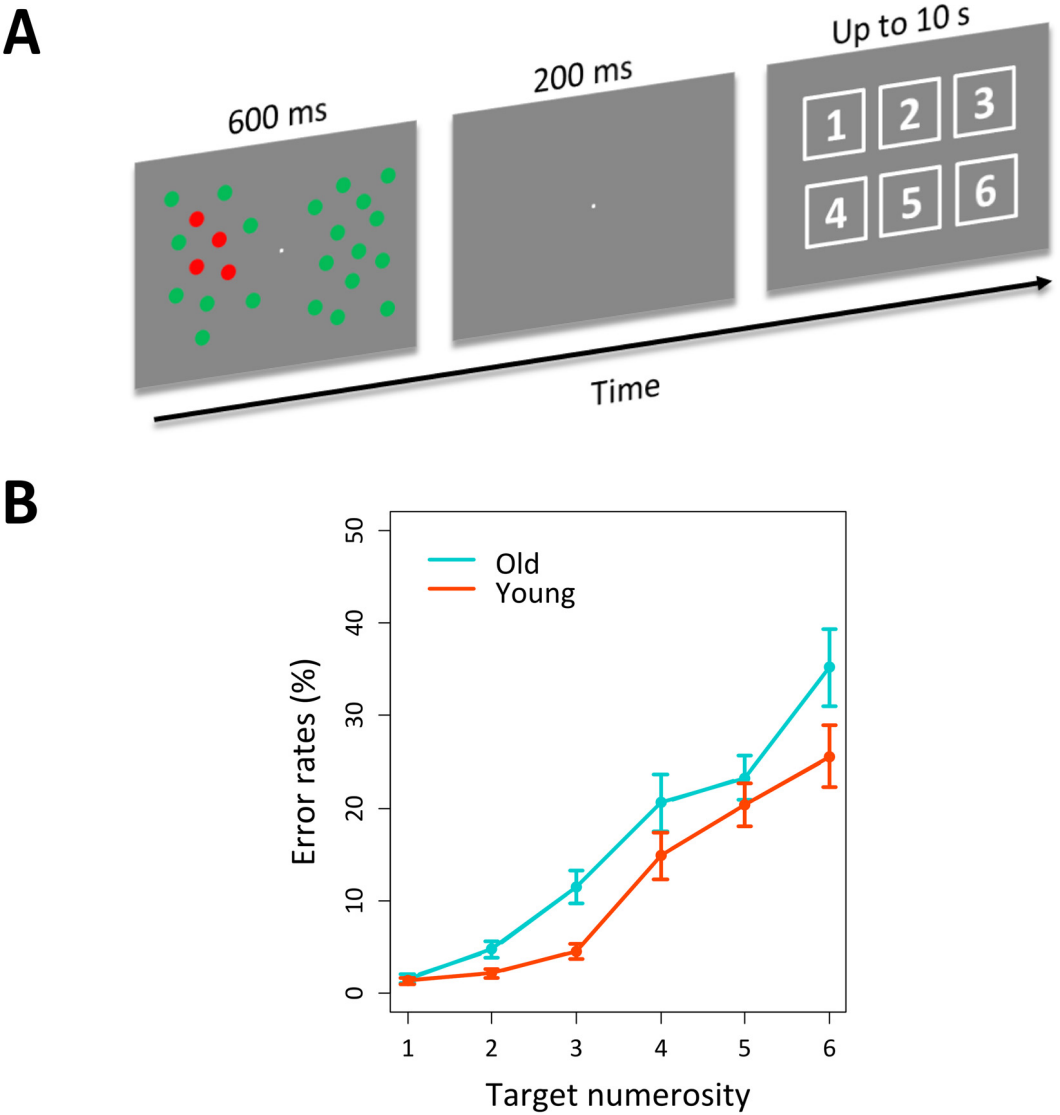
Stimulus example and accuracy results. (A) Example of a trial with four targets Participants had to indicate the number of targets by clicking on one of the six squares in the response display. (B) Accuracy results (error rates) as a function of age group and numerosity.

Stimuli consisted of 24 equiluminant red (35 cd/m^2^) and green (35 cd/m^2^) dots (0.97°) presented on a dark gray background (22 cd/m^2^) and equally distributed to the right and left of a white fixation dot (0.2°). The dots were displayed within an invisible grid of 8 rows x 10 columns (13.8° x 16.4°) centered on the fixation. In each trial a variable number (from 1 to 6) of uniquely colored targets (either red or green, counterbalanced across participants) was presented either to the left or to the right of the fixation, in random order. Targets never appeared in the extreme columns and rows of the matrix, or in the columns adjacent to the fixation. Participants’ task was to report the number of targets using the computer mouse. The response was provided by clicking one out of six squares displaying the Arabic digits from 1 to 6 (see Fig 1A).

Participants sat in a dimly lit room at approximately 85 cm from a computer screen. Each trial began with an interval (from 1480 to 1520 ms) displaying the fixation point. The stimulus configuration was displayed for 600 ms. Participants’ task was to count the number of targets while keeping their eyes on the fixation point. After an additional blank frame (200 ms) the response screen was displayed for a maximum time of 10 s. Participants provided their response by clicking one of the 6 squares presented on the screen. A total of 960 trials (16 blocks, 160 trials for each level of numerosity) was delivered to participants. A practice block of 16 trials was delivered prior to the experimental session. The old group performed an additional task (32 trials) prior to the experimental session to familiarize themselves with the use of the mouse. The task was the same as the experimental task but stimuli consisted of white Arabic digits presented at the center of the screen. Participants reported the identity of the number by clicking on one of the six squares of the response screen, as in the experimental task.

### EEG recordings and ERP analysis

Electroencephalogram (EEG) was recorded and digitized at 1 kHz from 32 active Ag/AgCl channels (including PO7 and PO8). The signal was referenced online to the right mastoid, and re-referenced off-line to the average of the left and right mastoids. The horizontal electro-oculogram (HEOG) was recorded from two electrodes placed at the canthi of both eyes. The impedance was kept on average below 10 kΩ for all channels. The signal was recorded with an on-line band-pass filter of 0.01 Hz- 250 Hz, and filtered offline with a low-pass filter of 40 Hz. Ocular artifacts (eye blinks and saccades) were individuated using an Infomax Indipendent Component Analysis (ICA) algorithm applied over 5 minutes of continuous EEG recording. Components representing eye blinks and saccades were visually inspected and discarded (the average number of discarded components was 2.1 in old adults and 1.9 in young adults) and the whole signal was then reconstructed using an inverse ICA algorithm. After this procedure, the EEG was segmented in 800-ms long epochs, starting 200 ms before stimulus onset, separately for each target numerosity (from 1 to 6 targets). Epochs containing other artifacts, such as head movements or muscular activity, were discarded using an amplitude threshold of ±80 μV on all channels except for the HEOG.

Mean difference amplitudes at PO7/8 were computed by subtracting the ipsilateral mean activity from the contralateral one, with respect to the side of target presentation and separately for each condition. The contralateral and ipsilateral amplitude values were obtained by collapsing the activity of the electrodes across target sides (i.e. PO7 was contralateral for right targets and ipsilateral for left targets and vice versa for PO8). Statistical analyses focused on two ERP components: N2pc (200–300 ms) and CDA (400–600 ms). For the N2pc only we also tested differences in peak latency and we thus extracted for each condition and each group the maximal negative peak over difference waveforms (contralateral minus ipsilateral differences) in the time window starting 150 ms post stimulus up to 300 ms post stimulus. After identifying the peaks we extracted latency and amplitude of each peak in each condition and group.

Behavioral and ERP data (N2pc: mean amplitude, peak latency and peak amplitude; CDA: mean amplitude) were analyzed using a mixed ANOVA with group (2 levels: young Vs. old participants) and numerosity (6 levels: 1–6 targets) as factors. The *p* values were corrected using the Greenhouse-Geisser method when violation of the sphericity assumption occurred and only corrected values are reported. Post-hoc analyses were carried out by means of polynomial contrasts (see S1 File for the full dataset).

### Alpha Event-related synchronization (ERD)

In order to examine posterior alpha lateralization in the old and young groups we extracted event-related synchronization/desynchronization (ERS/ERD) measures in the alpha band (8–15 Hz). ERS/ERD measures allow to visualize variation of a frequency band with respect to a prestimulus baseline as a function of time [32,33]. The results of the ERS/ERD calculation yields values that are either positive, meaning an increase in the power of the frequency band with respect to a baseline or synchronization, or negative, meaning a decrease in power or desychronization. To compute time-frequency values expressing changes with respect to the baseline we used the event-related spectral perturbation method (ERSP) [41]. We thus extracted artifact-free epochs starting 500 ms before stimulus onset and lasting for 1.5 sec. Next, we downsampled at 500 Hz and applied on each epoch a complex Morlet wavelet transform that increased linearly from 1.5 cycle at 4 Hz to 7 cycles at 30 Hz. The maximal wavelet length was 418 ms at 4 Hz. Epochs were zero-padded to a power of 8 to increase frequency resolution. The procedure yielded time-frequency spectrograms with a time window starting 291 ms before stimulus onset to 789 ms post stimulus. We averaged time-frequency values in the alpha band (8–15 Hz) separately for numerosity (6 levels), group (2 levels) and laterality (ipsilateral Vs contralateral) to obtain alpha ERS/ERD time course. The statistical analyses focused on the same time window of the ERPs (0–600 ms post stimulus). To evaluate the time course of alpha ERD we further divided the interval in two time windows of 300 ms each (0–300 ms and 300–600 ms). We submitted the mean values obtained for each time window to a mixed ANOVA using the following factors: time (2 levels: early and late), numerosity (6 levels 1–6), group (2 levels: old and young) and laterality (2 levels: ipsi and contralateral).

## Results

### Behavioral

An ANOVA was conducted on mean error rates. The group of old participants performed worse than the group of young participants, as indicated by a significant effect of group (*F*(1,34) = 4.8, *p* <.05). This effect was coupled with a general increase in error rates as a function of target numerosity (main effect of numerosity: *F*(5,170) = 76.8, *p* <.001; see Fig 1). To better understand the effect of numerosity on error rates we further computed post-hoc polynomial contrasts. In line with the results on the subitzing effect, the analysis showed a significant quadratic trend *F*(1,17) = 6.4, *p* <.05, with the percent of errors starting to increase more steeply for more than 3 targets (see Fig 1). Taken together the data showed a general reduction of the ability to enumerate targets among distractors in the old participants.

### ERPs

#### N2pc

**Fig 2:**
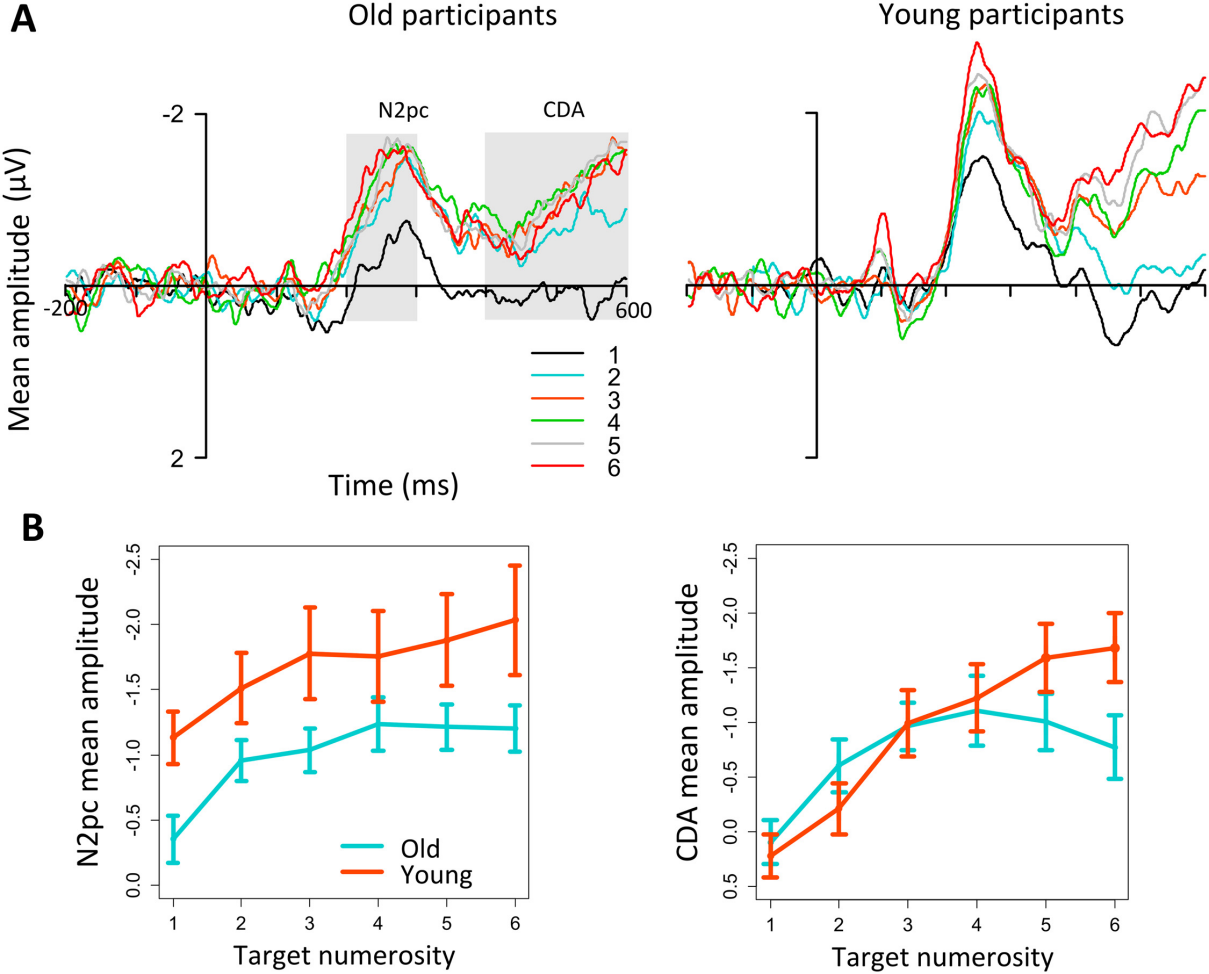
Event related potential results. (A) ERP difference waveforms as a function of age group and numerosity. Panels represent respectively numerosities 1 to 6 for the old group group (left) and for the young group (right). (B) Mean amplitude values for the N2pc (200–300 ms) and CDA (400–600 ms) as a function of age group and numerosity.

Overall, the mean amplitude of N2pc was significantly suppressed in the old group with respect to the young group, as indicated by a significant main effect of group (*F*(1,34) = 4.1, *p* <.05). N2pc mean amplitude increased as a function of target numerosity in both young and old participants, leading to a significant main effect of numerosity (*F*(5, 170) = 11.78, *p* <.001). As for the behavioral data, we conducted *post-hoc* polynomial contrast tests. In line with previous studies [9,11,12] the contrast test showed a significant quadratic trend *F*(1,17) = 1.4, *p* <.01, suggesting that N2pc amplitude increased up to three elements (see Fig 2). The ANOVA on N2pc peak amplitude values indicated the same pattern of results (main effect of group: *F*(1,34) = 5,7 *p* <.05; main effect of numerosity: *F*(5,170) = 13.1, *p* <.01).

The results of the analysis on N2pc peak latency showed no significant effects, all *p*s>.12.

#### CDA

The amplitude of the CDA was modulated by target numerosity in both groups, as shown by a significant main effect of target numerosity (*F*(5,170) = 27.2, *p* <.001). Differently from N2pc, the CDA was similar in the two groups for small target quantities, whereas it increased more steeply for the young than for the older group with larger numerosities, as showed by a significant interaction between group and numerosity (*F*(5,170) = 4.6, *p* <.01; see Fig 2). Since the interaction was significant, we computed the post-hoc polynomial contrasts separately for each group. The results showed that while the young group showed a significant linear trend *F*(1,17) = 49.5, *p* <.01, the old group showed a significant quadratic trend *F*(1,17) = 36.6, *p* <.01, meaning that CDA reached a plateau at approximately three targets in the old group but continued to increase above three targets in the young group (see Fig 2).

#### ERS/ERD

**Fig 3:**
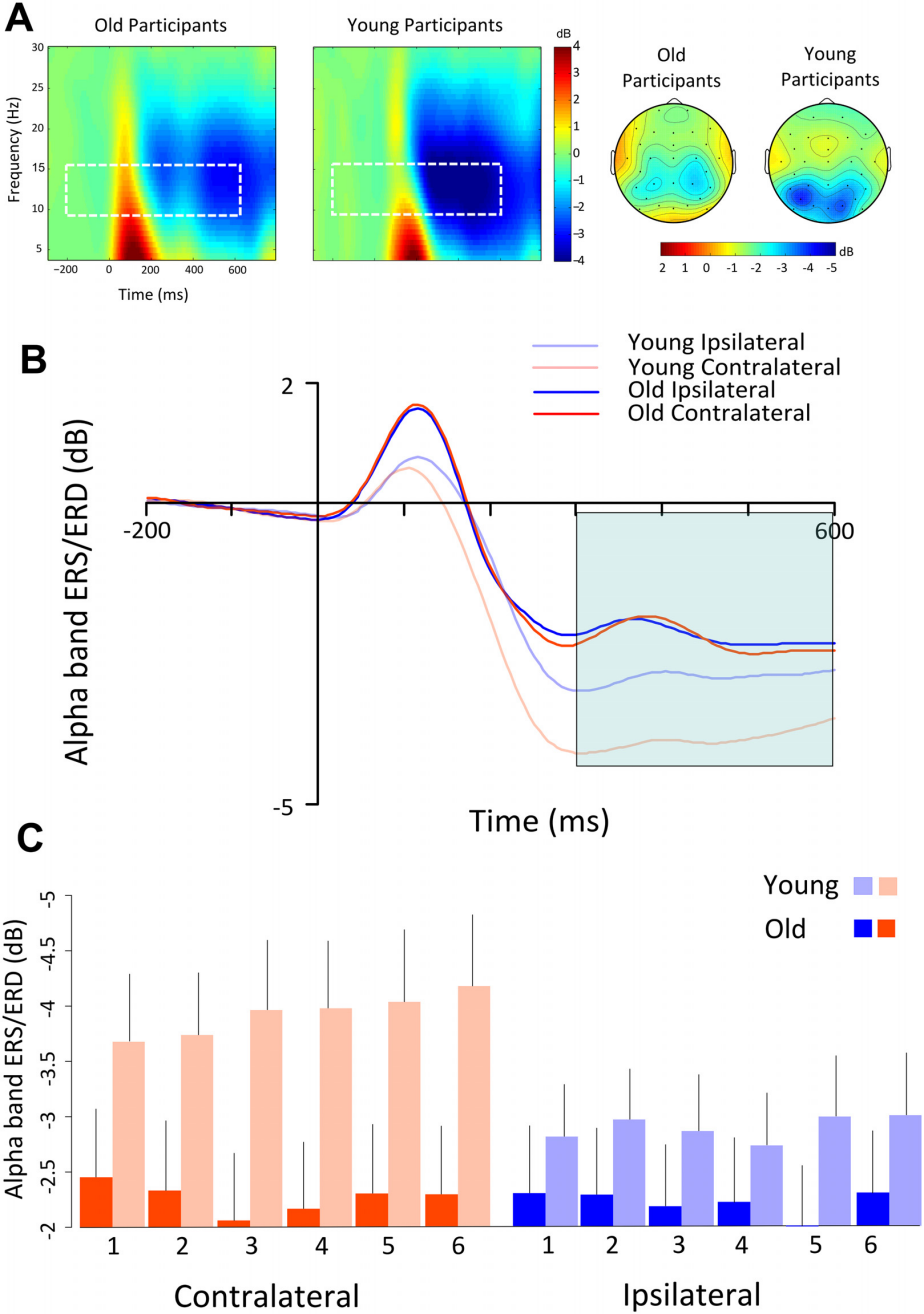
Time/frequency analysis results. (A) Left: Time-frequency plots for the old and young group averaged across numerosities and side at PO7/8. The white dotted squares indicate the time-frequency bins used to analyze ERS/ERD in the alpha band. Right: Topographical maps of the alpha band in the 300–600 ms post-stimulus window plotted separately for the old and young group and collapsed across numerosities. Contralateral side is plotted on the left. (B) Posterior (PO7/8) ipsilateral and contralateral ERS/ERD time course averaged across numerosities in the old and young group in the alpha band. The young group shows a significant alpha lateralization effect starting around 300 ms post stimulus. (C) Mean alpha ERD as a function of numerosity, laterality and age in the 300–600 time window.

The results of the ANOVA on ERS/ERD using time, group, numerosity and laterality as factors showed significant effects of laterality, *F*(1,34) = 4.2, *p* <.05, time, *F*(1,34) = 92.6, *p* <.001, time X laterality *F*(1,34) = 4.1, *p* <.05, group X numerosity X time *F*(5,170) = 3.6, *p* <.01, and group X numerosity X time X laterality, *F*(5,170) = 2.2, *p* <.05. Separate ANOVAs were conducted separately for each time range. The ANOVA for the 0–300 ms time window showed no significant effects (all *p*s>.13). The ANOVA in the 300–600 ms time window indicated significant effects of laterality *F*(1,34) = 5,4, *p* <.05, group X numerosity *F*(5,170) = 2.4, *p* <.01, group X laterality *F*(1,34) = 3.4, *p* <.05, and group X laterality X numerosity, *F*(5,170) = 3.7, *p* <.01. Follow-up ANOVAs were conducted separately for each group. In the young group we found a significant main effect of laterality *F*(1,17) = 7.9, *p* <.01 and a significant interaction between laterality and numerosity *F*(5,85) = 2.6, *p* <.05. There was a significant effect of numerosity *F*(5,85) = 3.3, *p* <.01 with a significant linear trend *F*(1,17) = 11.1, *p* <.01 for the contralateral electrodes. No significant effect of numerosity was present for the ipsilateral electrodes (*F*<1, *p* = .56). Taken together, these results indicate a larger decrease in alpha power for contralateral than ipsilateral electrodes in young adults, and that this lateralized decrease was monotonically modulated by numerosity (see Fig 3). In contrast, no significant effects emerged for the old group (all *p*s>.10).

## Discussion

The results of the present study shed new light on the nature of the age-related decline during enumeration by pointing out a joint involvement of both early attention individuation and late working memory procedures.

The behavioral results showed that the enumeration performance was overall impaired in old participants, replicating previous results on target enumeration and aging in cluttered scenes [3]. In previous studies, this effect was explained in terms of a reduction in the attentional resources dedicated to the task. Our electrophysiological approach extends this previous interpretation by pointing out the involvement of both attention and VWM in age-related changes during enumeration.

The results on N2pc showed that in both groups this component was modulated by target numerosity and reached a plateau at approximately three targets, in line with previous studies on young adults [9,11,12,22] indicating a link between the amplitude and the modulation of N2pc and the ability to enumerate small quantities of objects [9,11,12]. However, the amplitude reduction of the N2pc component in old individuals suggests that the attention mechanism reflected by the N2pc is overall less efficient in aging for all the target numerosities used here. The reduction of N2pc is found in the group of participants who also have a reduction in performance, as showed by the behavioral results. This result supports the proposal of Watson and colleagues suggesting that attentional resources during enumeration are reduced in aging [3,42]. In addition, the pattern replicates the results of a previous study on multiple object tracking [15], suggesting that the decline in target individuation due to aging affects the execution of a variety of tasks requiring multiple object processing.

The results on CDA provided additional information on the role of VWM in visual enumeration during aging. As for the N2pc, the CDA amplitude was modulated by target numerosity in both age groups, in line with previous results on young adults engaged in visual enumeration tasks [11,12]. However, differently from N2pc, the CDA was not suppressed over the entire numerosity range in the old group. For target numerosities within the subitizing range (1–3) the CDA amplitude did not differ between young and old adults. The difference between the two groups emerged only for the large numerosity set, with overall larger amplitudes for young than old participants. Thus, in contrast with the N2pc pattern, the CDA results indicate that for small target numerosities (1–3 targets) the VWM procedures used by old adults are as efficient as the ones used by their younger counterparts. Together with the N2pc findings, they also suggest that VWM can act as a support for the reduction of attentional resources deployed toward multiple objects, at least for small target quantities (1–3 targets). In contrast, the difference between the two groups for the largest numerosities (4–6 targets) likely reflect the ability of young adults to maintain in memory (at least on some trials) more than three targets. This would in turn explain the overall low error rate of the young adults in the present study as compared with previous ones [11,12].

Two interesting aspects emerged from the analysis of alpha lateralization. First, we found a significant modulation of alpha ERD as a function of target numerosity in young adults. This effect occurred in the late time window (300–600 ms), and is in line with the interpretation of numerosity-related modulation of alpha oscillations as reflecting the functioning of working memory procedures [43,44]. The modulation was visible in contralateral electrodes, thus suggesting that the effect is relative to the processing of the elements in the relevant hemifield (for different results, see [45]). The similarity between the alpha and CDA patterns found in the present study further suggests a link between these two lateralized neural effects [46]

Second, and in contrast with the results on young individuals, the results indicate that the alpha modulation of target numerosity was absent in old participants. More notably, even the overall lateralization of alpha ERD was strongly attenuated in old adults [47], possibly indicating a deficit in the ability to modulate the activation of the two visual hemispheres as a function of the side of presentation of the relevant (target) objects (for a similar effect on pre-stimulus activity see [48]). Overall, these results suggest a decline in the processing resources for the target elements in aging. In conclusion, our study indicates that changes in various neural responses associated with both attention and working memory contribute to the age-related decline in enumeration abilities. The behavioral impairment in aging is associated with a less functional attentional individuation stage, as indexed by the N2pc pattern, and with differences in VWM procedures (as reflected by the CDA modulation) that occur only for large target numerosities. The analyses of alpha posterior ERD lateralization provide converging evidence of a decreased activity in the visual cortices contralateral to the target hemifield during processing of multiple targets in aging.

## Supporting Information

S1 File Data used for the analyses of the paper “Electrophysiological correlates of subitizing in healthy aging”: Preprocessed data including accuracy, ERPs and ERS/ERD values. (XLSX)
